# Dual-Guided Brain Diffusion Model: Natural Image Reconstruction from Human Visual Stimulus fMRI

**DOI:** 10.3390/bioengineering10101117

**Published:** 2023-09-24

**Authors:** Lu Meng, Chuanhao Yang

**Affiliations:** College of Information Science and Engineering, Northeastern University, Shenyang 110819, China; 2100877@stu.neu.edu.cn

**Keywords:** visual reconstruction, diffusion model, brain decoding, fMRI

## Abstract

The reconstruction of visual stimuli from fMRI signals, which record brain activity, is a challenging task with crucial research value in the fields of neuroscience and machine learning. Previous studies tend to emphasize reconstructing pixel-level features (contours, colors, etc.) or semantic features (object category) of the stimulus image, but typically, these properties are not reconstructed together. In this context, we introduce a novel three-stage visual reconstruction approach called the Dual-guided Brain Diffusion Model (DBDM). Initially, we employ the Very Deep Variational Autoencoder (VDVAE) to reconstruct a coarse image from fMRI data, capturing the underlying details of the original image. Subsequently, the Bootstrapping Language-Image Pre-training (BLIP) model is utilized to provide a semantic annotation for each image. Finally, the image-to-image generation pipeline of the Versatile Diffusion (VD) model is utilized to recover natural images from the fMRI patterns guided by both visual and semantic information. The experimental results demonstrate that DBDM surpasses previous approaches in both qualitative and quantitative comparisons. In particular, the best performance is achieved by DBDM in reconstructing the semantic details of the original image; the Inception, CLIP and SwAV distances are 0.611, 0.225 and 0.405, respectively. This confirms the efficacy of our model and its potential to advance visual decoding research.

## 1. Introduction

Mind reading has been a captivating concept often depicted in movies, and recent advancements in deep learning technology have brought us closer to the possibility of interpreting brain activity. The human visual system, as a critical organ for perceiving external information, has been a central focus of neuroscience research, particularly in functional studies. The investigation of brain vision can be broadly categorized into two distinct tasks: visual encoding and visual decoding. Visual encoding involves the transformation of external visual stimuli into neural activity signals in the brain, which aids in understanding the brain’s mechanisms of encoding visual information and also contributes to the advancement of machine vision research. On the other hand, visual decoding aims to extract characteristic information of visual stimuli from collected brain activity signals, such as location, direction, and stimulus category [1,2,3,4]. Visual decoding can further be classified into three sub-tasks: classification, recognition, and reconstruction [5]. Among these, visual reconstruction poses the most challenging problem, requiring the retrieval of all the information of the visual stimulus from brain activity. This task becomes particularly difficult due to the complexity of brain signal characterization and the inherent limitations of functional magnetic resonance imaging (fMRI) [6], such as its low signal-to-noise ratio, high dimensionality, and limited sample size, making the reconstruction of natural images perceived by the human brain an extremely challenging endeavor.

In the early stages, traditional visual image reconstruction methods predominantly relied on hand-made features [7] and regression models [8,9] to reconstruct simple geometries from fMRI signals [10,11,12]. Although these early explorations demonstrated the feasibility of decoding perceptual image semantic information from brain signals, the limitations of linear methods became apparent, resulting in reconstructions that were fuzzy and even lacking in meaningful content. The advent of deep learning methods, especially the emergence of advanced deep generative models, has revolutionized the field of visual decoding. Several studies have leveraged the powerful generative capabilities of large pre-trained networks, such as GAN [13,14], VAE [15,16], and diffusion models [17,18,19,20] to enhance the quality of reconstructed images, yielding impressive results. These models often involve mapping fMRI voxels to image feature spaces and fine-tuning pre-trained generative models to generate images based on predicted features. As a result, more complex visual stimulus reconstructions, including faces [21,22] and natural images [23] and complex scenes [24,25] have been successfully explored. Reconstruction methods for natural images can be broadly classified into two categories. The first category emphasizes achieving pixel-level similarity with the original image. For example, Shen et al. [26] integrated a deep generative network with a deep neural network (DNN) to optimize the pixel values of the input image using the DNN’s feature loss to generate a realistic reconstruction. Beliy et al. [27] constructed an encoding–decoding network based on a convolutional neural network (CNN), which was jointly trained on unpaired data to address the problem of scarcity in {fMRI image} samples. Gaziv et al. [28] further improved reconstruction quality by introducing perceptual loss for image reconstruction, building upon the work of Beliy et al. [27]. Ren et al. [29] proposed a dual VAE-GAN model and a three-stage learning strategy that combined adversarial learning and knowledge distillation. The second category of reconstruction methods aims to generate reconstructions that are semantically close to the stimulus image. Mozafari et al. [13] proposed the first semantic-related reconstruction model using BigBiGAN [30]. Ozcelik et al. [31] further advanced this approach by reconstructing images with accurate semantics from fMRI using Instance Condition GAN. Ferrante et al. [19] employed a latent diffusion model guided by text semantics to recover images that were perceptually similar to visual stimuli.

Although the above methods have achieved significant progress in visual reconstruction, they all suffer from certain limitations. For the approaches that focus on pixel-level similarity, their reconstructed images tend to be blurry and hardly identifiable. While those approaches emphasize generating semantically meaningful images, the obtained reconstructions often lack semantic consistency with the objects in the original images. Therefore, achieving the perfect reconstruction of visual stimuli remains to be explored continuously. Recent advancements in image generation models, notably the diffusion model, have demonstrated the capability to reconstruct intricate images with high resolution. Based on the above analysis, we propose a novel visual reconstruction framework, termed the Dual-guided Brain Diffusion Model (DBDM), which aims to reconstruct images with the original image’s semantic content and underlying features from brain activity. The DBDM model utilizes a Versatile Diffuser (VD) [32] with powerful generative capabilities, conditioning the image generation on both visual and semantic features from Contrastive Language–Image Pre-Training (CLIP) [33]. Specifically, we first utilize the Very Deep VAE (VDVAE) [34] to obtain a rough image representation of the visual stimulus. Subsequently, we employ the Bootstrapping Language–Image Pre-training (BLIP) [35] model to generate text descriptions for all training images in the fMRI dataset. Considering the complexity of characterizing brain signals, we design a deep neural network with residual connections (BrainMlp) as the neural decoder for the accurate estimation of visual and semantic features from fMRI data. Finally, the image-to-image pipeline in the pre-trained versatile diffusion model is utilized to accurately reconstruct perceptual images guided by the predicted visual and textual features and using the initial guess images generated in the first stage as input. Our contributions are summarized as follows:

- We introduce the Dual-guided Brain Diffusion Model (DBDM), which leverages the powerful generative capabilities of VD to reconstruct brain-perceived images that are semantically consistent with real images while retaining precise details, guided by both visual and semantic features;
- We generate text descriptions for each training image using BLIP to introduce semantic content. Additionally, we design the BrainMlp model with residual connections to learn the mapping of fMRI data to CLIP-extracted visual and semantic features, employing the well-trained model to predict the corresponding feature vectors from test fMRI data. Subsequently, we use the predicted visual and semantic features to modulate the inverse diffusion process of VD, providing sufficient guidance for reconstructing images similar to the original stimulus;
- We conduct comprehensive experiments on a publicly available dataset to evaluate the effectiveness of our proposed method. The experimental results demonstrate that DBDM achieves advanced results in both qualitative and quantitative comparisons with existing methods, enabling the reconstruction of high-resolution and high-fidelity images from fMRI signals.

## 2. Materials and Methods

This section presents an overview of the three phases of the Dual-guided Brain Diffusion Model (DBDM) and provides details regarding the dataset used in this study. The raw fMRI data used in the experiments are publicly available at https://openneuro.org/datasets/ds001246/ (accessed on 7 October 2022). The experiments were conducted on a server equipped with an NVIDIA 3090 GPU and 4 TB of RAM.

### 2.1. Dataset

For this study, we utilized the widely used Generic Object Decoding (GOD) dataset [11], which serves as a benchmark dataset for visual decoding research and stimulus reconstruction. The GOD dataset comprises fMRI recordings from 5 subjects during image presentation and imaging trials. All stimulus images presented in the dataset were randomly selected from the ImageNet database. The training set of the image presentation experiment consisted of 150 categories with 8 images per category, resulting in a total of 1200 stimulus images. Additionally, 50 images of different classes were chosen to form the test set. It is important to emphasize that the categories of the test set images do not overlap with those in the training set. During the image presentation experiments, two different acquisition schemes were employed: for the training data, each of the 1200 images was presented once, whereas for the test data, each of the 50 images was presented 35 times, with each stimulus image being presented for 9 seconds. To ensure subjects’ attention was focused on the presented images, they were asked to press a button when they saw two identical images. Moreover, the dataset includes masks for different regions of interest (ROI) to facilitate further analysis.

### 2.2. Overview

**Figure 1:**
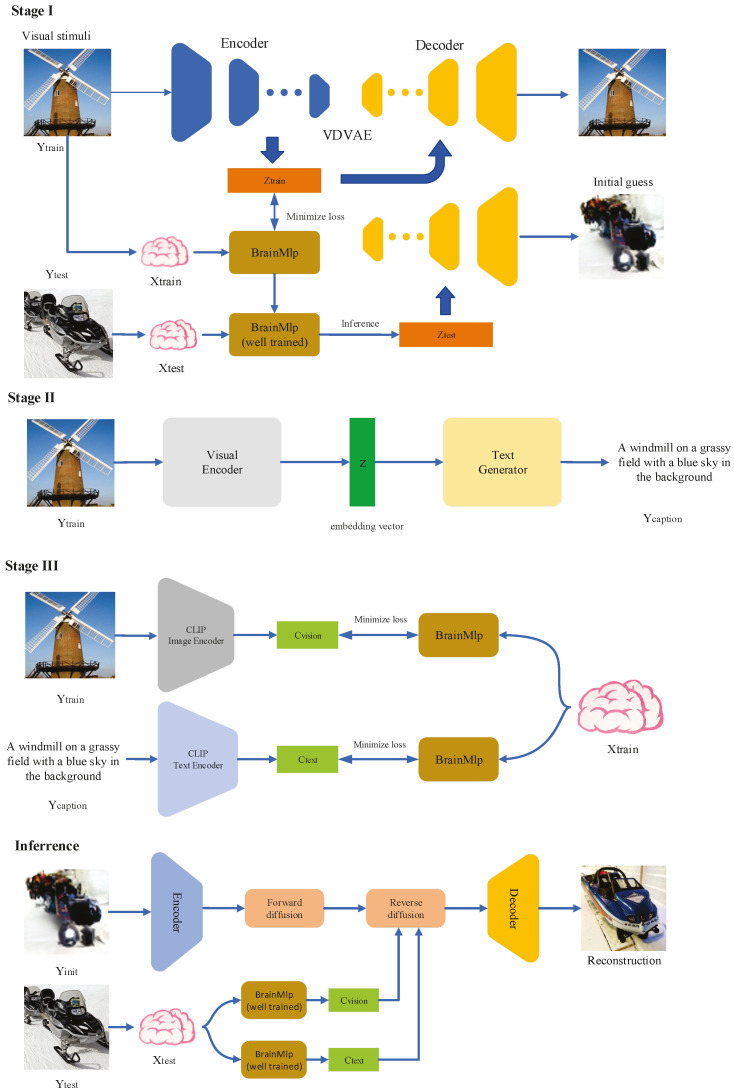
The overall reconstruction framework, which consists of three distinct stages.

We use {X, Y} to represent the {fMRI, Image} sample pairs. The training and test images are denoted by $Y_{train}$ and $Y_{test}$, respectively, and the fMRI training and test samples are denoted by $X_{train}$ and $X_{test}$, respectively. The reconstruction framework we propose is shown in Figure 1, which consists of three different stages: the initial guessing stage, the image caption generation stage, and the image reconstruction stage.

Initial guessing stage. First, $Y_{train}$ was fed into the encoder of VDVAE to extract the latent variables at different levels and connect them into a one-dimensional feature vector $z_{train}$. The BrainMlp model was then trained using {$X_{train}$, $z_{train}$} to learn the fMRI-to-latent-vector transformation, and the well-trained model was used to predict the latent vector $z_{test}$ based on $X_{test}$. Finally, $z_{test}$ was fed into the VDVAE decoder component to obtain the initial guess ${\hat{Y}}_{init}$ of the perceived image $Y_{test}$.

Image caption generation stage. We used the image caption generation pipeline in the BLIP pre-trained model provided by Li et al. [35] to generate text descriptions for the training images. Specifically, $Y_{train}$ was fed into the BLIP decoder to obtain the image caption $Y_{caption}$ with its visual encoding and text decoding modules.

Image reconstruction stage. We utilized the CLIP model trained on large-scale image-text paired data to extract the visual and textual features of the training images. As illustrated in Figure 1, stage III, the visual features $c_{vision}$ were obtained by feeding the training image $Y_{train}$ into the CLIP image encoder, and the semantic features $c_{test}$ were extracted by feeding the image caption $Y_{caption}$ into the CLIP text encoder. We constructed two different BrainMlp models to learn the mapping of $X_{train}$ to $c_{vision}$ and $c_{test}$, respectively, and then used the well-trained models to predict the corresponding ${\hat{c}}_{vision}$ and ${\hat{c}}_{test}$ based on the fMRI test pattern $X_{test}$. In the generation stage, we took the initial guess image ${\hat{Y}}_{init}$ generated by VDVAE as the input of the VD image-to-image pipeline to obtain the latent vector through the AutoKL encoder and then performed a diffusion process on this latent vector. The resulting latent noise vector was employed as the initial noise of the reverse diffusion process, and the ${\hat{c}}_{vision}$ and ${\hat{c}}_{test}$ obtained above were used as conditional guidance. Finally, the obtained denoised vector was used as the input of the AutoKL decoder to generate the final reconstruction ${\hat{Y}}_{test}$.

### 2.3. Stage I: Obtain the Initial Guess Using VDVAE

VAE is actually a variant of Autoencoder (AE), and the motivation for its use is to transform AE into a generative model. In VAE, we need to model the true distribution $p\left(x\right)$ of the data in order to generate new samples. VAE avoids the challenge of directly modeling $p\left(x\right)$ and instead constructs a transformation from the prior distribution $p\left(z\right)$ of a given hidden variable to the distribution $p\left(x\right)$ of the real data. For this, we can model $p\left(x|z\right)$ with a decoder. When the model is well-trained, a new sample can be generated by sampling on the prior Gaussian distribution $p\left(z\right)$ and then entering the result into the decoder. Although VAE is theoretically capable of modeling any complex data distribution, it is practically impossible to characterize $p\left(x\right)$ perfectly due to computational constraints and optimization difficulties. In particular, when the input data have a complex distribution, latent variables with more complex distributions are required; thus, a simple VAE is not enough. In order to alleviate this problem, hierarchical VAE (HAVE) has been proposed to increase the expression ability of the approximate posterior distribution and prior distribution. Its hierarchical dependencies are shown in the following formula.

(1) $$p_{\theta }\left(z\right)=p_{\theta }\left(z_{1}\right)\prod_{i=2}^{L}p_{\theta }\left(z_{i}|z_{<i}\right)$$

(2) $$q_{\phi }\left(z|x\right)=q_{\phi }\left(z_{1}|x\right)\prod_{i=2}^{L}q_{\phi }\left(z_{i}|x,z_{<i}\right)$$

(3) $$p_{\theta }\left(x|z\right)=p_{\theta }\left(x|z_{L}\right)p_{\theta }\left(z\right)=p_{\theta }\left(x|z_{L}\right)p_{\theta }\left(z_{1}\right)\prod_{i=2}^{L}p_{\theta }\left(z_{i}|z_{<i}\right)$$

where $z=\left(z_{1},z_{2},\ldots ,z_{L}\right)$ denotes the latent vectors, which have different dimension sizes; e.g., $z_{1}$ has a lower dimension (corresponding to the top feature map of the network), and $z_{L}$ has a higher dimension (corresponding to the bottom feature map of the network). *x* is the input variable, $q\left(z|x\right)$ denotes the approximate posterior distribution learned by the encoder, and $p\left(z\right)$ denotes the prior distribution.

In this research, we employed the Very Deep Variational Autoencoder (VDVAE) [34], which consists of 75 layers and was pretrained on the ImageNet dataset [36] with image sizes scaled to 64×64, as the generative model for initial image estimation. The VDVAE utilizes a hierarchical VAE architecture and addresses the issues of instability and high computational cost in HVAE training. For the purpose of initial image estimation, we utilized the hidden variables of the first 31 layers as the encoding features for the image, as these latent variables are considered sufficient to adequately encode complex natural images. As illustrated in Figure 1, Stage I, the fMRI training set images were fed into the encoder of VDVAE to extract latent vectors from various layers. Given that the latent vectors from different layers have distinct dimensional sizes, we combined the latent vectors of the first 31 layers to create a 91168-dimensional feature vector. Subsequently, we utilized the BrainMlp model to learn the mapping of fMRI voxels to their corresponding feature vectors. During the inference phase, the test fMRI samples were inputted into the trained BrainMlp to predict the feature vectors corresponding to the stimulus images, and the predicted vectors were reshaped into latent variables of different layers of VDVAE. These reshaped latent variables were then fed into the decoder of VDVAE to obtain the initial reconstructed images.

### 2.4. Stage II: Generate Text Description Using BLIP

In the realm of human life, textual descriptions are commonly employed to convey visual information, serving as a significant complement to image features. Hence, the incorporation of textual descriptions of images within the reconstruction framework proves beneficial in enhancing the quality of the reconstructions. Moreover, recent research has demonstrated that large language models exhibit a certain correlation with brain activity signals, rendering them suitable for neural decoding tasks [37]. However, the visual stimuli used in the Generic Object Decoding (GOD) dataset are derived from ImageNet and lack corresponding textual descriptions. To address this, we employed the state-of-the-art (SOTA) Vision and Language Pre-training (VLP) model known as BLIP to generate captions for the training images, as depicted in Figure 1, Stage II. The BLIP model integrates visual–language understanding and generation tasks, achieving SOTA performance in image caption generation through the utilization of the MED structure and CapFilt data augmentation approach. The BLIP model employs a visual transformer [38] as its image encoder, encoding the input image into embedding sequences. Subsequently, it generates a corresponding text description based on these embeddings via a text decoder. The generated image captions are employed in the subsequent stage to extract semantic features, thereby guiding the diffusion model in generating reconstructions that are semantically meaningful.

### 2.5. Stage III: Image Reconstruction

As illustrated in Figure 1, Stage III, the initial image reconstructed by VDVAE captures the layout information of the original image but may lack high-level characteristics, leading to challenges in recognizing the image content. To refine the results further and obtain a final reconstruction, we utilized the recently proposed Latent Diffusion Model (LDM). The LDM effectively guides the inverse diffusion process through visual and semantic representations, resulting in high-quality reconstructions with semantic consistency and faithful low-level details akin to the original image. We took the initial guess of the brain-perceived image obtained in Stage I as input to the Versatile Diffuser (VD) image-to-image pipeline, encoding it as latent variables. Subsequently, by progressively introducing noise to the latent variables, we obtained the latent noise vector, which served as the initial noise for the VD. This approach enables the constraint of the position and shape of objects in the generated image, promoting greater consistency with the stimulus image while ensuring high-quality reconstruction.

Incorporating visual and semantic guidance, we employed the visual encoder and text encoder within the pre-trained CLIP model to extract CLIP visual features and CLIP text features of the stimulus images, respectively. Two BrainMlp decoders were then trained: a visual decoder and a semantic decoder, which were responsible for learning the mapping of fMRI voxels to CLIP visual features and CLIP semantic features, respectively. During the inference phase, the initial noise was utilized as input, guiding the denoising process of the diffusion network based on the CLIP visual and CLIP semantic features predicted by the decoders. Finally, the latent denoising vector was fed into the Autoencoder’s decoder to invert it into an image, yielding a high-quality reconstruction.

### 2.6. Statistical Analysis

In this section, we present a comprehensive overview of the statistical analysis conducted to rigorously assess the performance of our novel visual decoding approach, the Dual-guided Brain Diffusion Model. While our study does not involve the comparison of distinct groups, we employed a set of key statistical metrics to quantitatively evaluate the effectiveness of our method. These metrics encompass:

- Pixel-wise correlation (PixCorr): This metric is used to measure the pixel-level similarity between the reconstructed and original images.
- Structural similarity index measure (SSIM): This provides a metric for the structural similarity between the reconstructed and original images.
- Inception distance: This metric evaluates the quality of generated images through feature space statistics.
- CLIP distance: This metric assesses the consistency between the reconstructed images and textual descriptions using the CLIP model.
- SwAV distance: This metric quantifies the alignment of image embeddings with respect to semantic content using SwAV.

To provide a holistic view of our approach, we compared the results obtained using DBDM with those achieved by the state-of-the-art (SOTA) methods in the field. This comparative analysis allows us to evaluate the relative performance of DBDM and its contributions to advancing visual decoding research.

The outcomes of our statistical analysis, in conjunction with the SOTA comparison, offer valuable insights into the performance of our DBDM model. This comparative evaluation highlights the strengths and advantages of our approach in relation to existing methods, reinforcing its potential to advance the state of the art in visual decoding from fMRI data.

## 3. Results

### 3.1. Implementation Details

The Versatile Diffuser (VD) utilized in this study was trained on the Laion2B-en dataset [39] with the inference step set to 50; the generated image size was set to 512×512. The CLIP network architecture employed to extract image visual features and text features was ViT-L/14, which leverages a substantial amount of image–text data for comparative learning. The dimension of the visual features extracted by CLIP is 257×768, while the text features of the subtitles are 77×768. Moreover, the intensities of visual and semantic guidance were set to 0.7 and 0.3, respectively. The pre-training weights of the BLIP model can be accessed at https://github.com/salesforce/BLIP (accessed on 8 June 2023).

**Figure 2:**
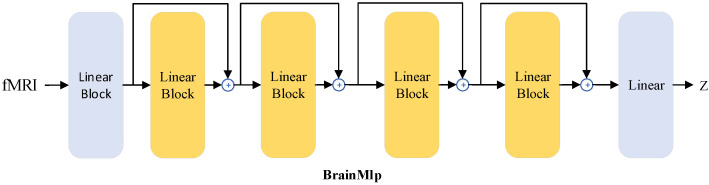
The structure diagram of BrainMlp.

BrainMlp, a deep network with residual connections, is composed of fully connected layers, as depicted in Figure 2. The “linear block” consists of a linear layer, LayerNorm layer, GELU activation layer, and Dropout layer. During the process of training BrainMlp, the AdamW optimizer was employed with an initial learning rate of $1\times 10^{-4}$. The learning rate is an important hyperparameter; when it is large, the model will find it difficult to converge to the optimal solution, but when it is small, the model will converge slowly. Experimental results indicate that our model can converge to the loss function minimum faster when the initial learning rate is $1\times 10^{-4}$. In addition, we introduced a cosine annealing learning rate decay strategy, where the learning rate decreases as the number of iterations increases, which ensures that the model does not fluctuate dramatically during the training process and thus is closer to the optimal solution. The batch size was set to 64, and the training process spanned 240 epochs. The loss function used in the training was a combined loss of mean squared error and cosine similarity.

(4) $$L_{mlp}=\alpha_{1}\frac{1}{N}\sum_{i=1}^{N}{∥z_{i}-{\hat{z}}_{i}∥}_{2}^{2}+\alpha_{2}\frac{1}{N}\sum_{i=1}^{N}\cos∠\left(z_{i}-{\hat{z}}_{i}\right)$$

where *N* is the number of samples, $z_{i}$ is feature vector, and ${\hat{z}}_{i}$ is the prediction result of BrainMlp. We set $\alpha_{1}=0.9$ and $\alpha_{2}=-0.1$.

Prior to utilizing the feature vectors ($z_{test}$, ${\hat{c}}_{vision}$, and ${\hat{c}}_{text}$) predicted by BrainMlp, a renormalization trick was applied. Specifically, we computed the mean and standard deviation of the extracted feature sequences from the training images. These statistics were then employed to replace the mean and standard deviation of the predicted features, facilitating normalization and thereby bringing the predicted features closer to the true feature distribution. This renormalization process aims to enhance the accuracy and reliability of the predicted features, aligning them more effectively with the underlying feature distribution of the training data.

### 3.2. Examples of Visual Reconstruction

**Figure 3:**
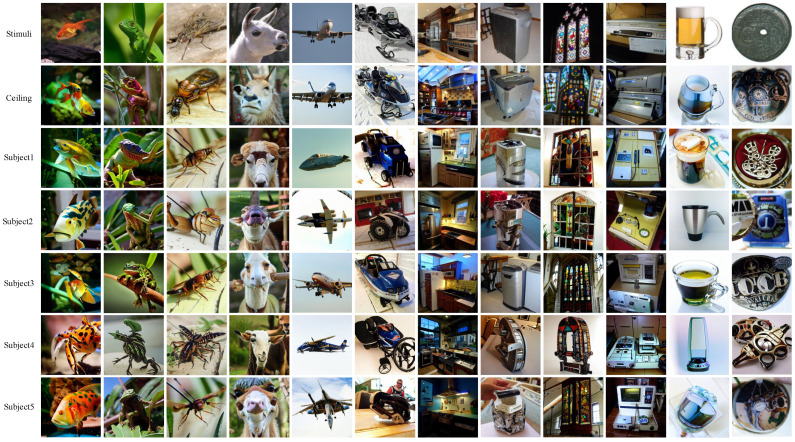
Perceptual images reconstructed by DBDM. The first row is the real visual stimulus. The second row is the ceiling of the model. The remaining rows are the reconstructed results for different subjects.

We present several reconstruction examples of our proposed Dual-guided Brain Diffusion Model (DBDM) in Figure 3. The first row showcases real stimulus images, while the second row illustrates the reconstructed images using ground-truth $c_{vision}$ and $c_{text}$ as conditional guidance, representing the optimal performance achievable by our model. The subsequent five rows exhibit reconstructions obtained from fMRI recordings of different subjects. As our method emphasizes reconstructing images that are semantically similar to the original stimuli, there might be some variations in pixel space. Nevertheless, owing to the dual guidance of vision and text and the provision of an initial guess image to constrain the generated image’s randomness, the details of the objects in the original images are predominantly preserved.

For instance, in the fifth column, our method accurately reconstructs images with the semantic meaning of an airplane, maintaining semantic consistency across all subjects, which stands as a noteworthy achievement. Although the reconstructed aircraft may exhibit some variations, their positions and layout within the images remain similar; they are typically centered in the picture with the sky as the background. Similarly, observations of the first to fourth columns reveal that when the object in the original image is an animal, all reconstructions also represent animals. This observation indicates that DBDM effectively captures high-level semantic information from the stimuli. Furthermore, we notice that the images reconstructed by DBDM also successfully retain the shape, contour, and other low-level details of the real image. For example, the shape of the reconstructed image in the twelfth column is consistent with the real image (all circular contours) and the texture is also restored. For the reconstructed image in the eighth column, similar outlines and colors to the original image are retained. In summary, our proposed method adeptly preserves low-level details while generating semantically meaningful images. Through the visual comparisons in Figure 3, we can observe that the reconstructions obtained using the fMRI signals of subject 3 are more similar to the stimulus images in terms of semantics and low-level details, thus obtaining the best results.

### 3.3. Comparison with Other Methods

#### 3.3.1. Visual Comparison

**Figure 4:**
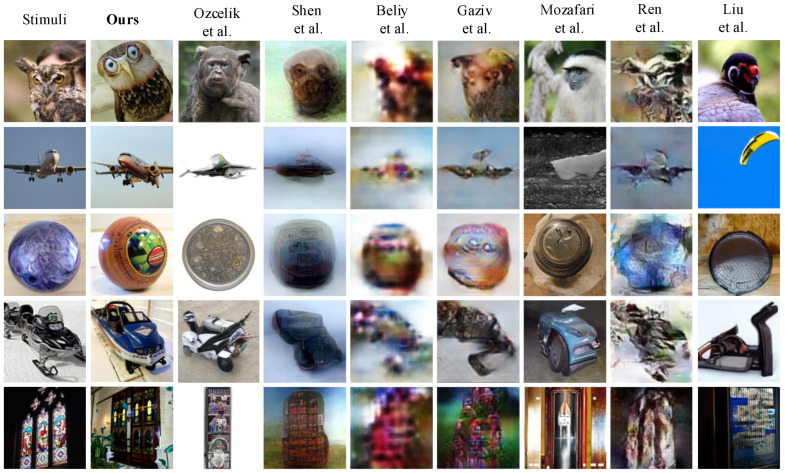
Visual comparison with different methods (Ozcelik et al. [31], Shen et al. [26], Beliy et al. [27], Gaziv et al. [28], Mozafari et al. [13], Ren et al. [29] and Liu et al. [20]). The images presented here were reconstructed using fMRI data of subject 3 because these data have the highest signal-to-noise ratio.

The reconstruction results of our proposed Dual-guided Brain Diffusion Model (DBDM) were visually compared with those of other advanced methods, as presented in Figure 4. For a fair and uniform comparison, we carefully selected the reconstructions provided by all the methods under evaluation. These methods can be categorized into two groups: those emphasizing pixel similarity (Shen et al. [26], Beliy et al. [27], Gaziv et al. [28], and Ren et al. [29]) and those focusing on semantic content matching (Ozcelik et al. [31], Mozafari et al. [13], and Liu et al. [20]).

As evident from Figure 4, the reconstructions produced by our method exhibit superior naturalness and semantic meaningfulness compared to those of Shen et al. [26], Beliy et al. [27], Gaziv et al. [28], and Ren et al. [29]. This distinction can be attributed to the fact that their approaches primarily concentrate on recovering objects with similar shapes, colors, and contours as the original images, often leading to blurry reconstructions and a lack of clear semantics. While Ozcelik et al. [31], Mozafari et al. [13], and Liu et al. [20] share similar objectives with our method, but by prioritizing the recovery of semantic content in the stimulus images, our approach achieves better semantic fidelity in reconstructing the original images and preserves more pixel-level details. For example, our method more accurately reconstructs an image of a large airplane flying in the sky compared to the methods of Ozcelik et al. [31], Mozafari et al. [13], and Liu et al. [20], which demonstrates the better semantic fidelity achieved by DBDM. For the bowling ball in the third row, DBDM more successfully reconstructs the spherical object compared to other methods, which simultaneously demonstrates the superiority of our method in reconstructing low-level features.

#### 3.3.2. Quantitative Comparison

To objectively compare our method with others, we employed five different evaluation indicators to assess the reconstruction quality, as displayed in Table 1. PixCorr was employed to measure the linear correlation between the reconstructed image and the original image in pixel space given two images, *X* and *Y*. It is computed as follows:

(5) $$\rho_{X,Y}=\frac{\mathrm{cov}(X,Y)}{\sigma_{X}\sigma_{Y}}$$

where $\sigma_{X}$, $\sigma_{Y}$ and cov(X,Y) are the standard deviation and covariance of *X*, *Y* respectively. SSIM is utilized to evaluate the structural similarity between images. It can be calculated using the following formula:

(6) $$SSIM=\frac{\left(2\mu_{X}\mu_{Y}+c_{1}\right)\left(2\sigma_{XY}+c_{2}\right)}{\left(\mu_{X}^{2}+\mu_{Y}^{2}+c_{1}\right)\left(\sigma_{X}^{2}+\sigma_{Y}^{2}+c_{2}\right)}$$

where $\mu_{X}$, $\mu_{Y}$, and $\sigma_{X}^{2}$, $\sigma_{Y}^{2}$ denote the mean and variance of *X* and *Y*, respectively. $\sigma_{XY}$ is the covariance, while $c_{1}$ and $c_{2}$ are constants. These two indicators were used to evaluate the low-level similarity between the reconstructions and the real images. Additionally, to evaluate semantic correlation in the reconstructed images, we employed three different networks (Inception-V3 [40], CLIPViT-B/32 [33] visual encoder, and SwAV-ResNet50 [41]) as feature extractors to calculate the distance between images in the feature space. The formula is as follows:

(7) $$d=1-\frac{\left(\mu -\bar{\mu }\right)\cdot \left(\nu -\bar{\nu }\right)}{∥\left(\mu -\bar{\mu }\right){∥}_{2}{∥\left(\nu -\bar{\nu }\right)∥}_{2}}$$

where μ and ν represent one-dimensional feature vectors and $\bar{\mu }$, $\bar{\nu }$ represent mean values. It is important to note that since not all methods reported these indicators, we recalculated them using the images provided in the respective papers. Furthermore, as different methods reconstruct the images in varying sizes, we scaled them to a uniform size during the indicator calculations. All metrics in Table 1 were calculated based on the reconstructed images of subject 3, as some authors only reported reconstructions for this specific subject.

As shown in Table 1, Ren et al.’s [29] method achieves the best performance on PixCorr due to the better preservation of color and texture in the original images. Gaziv et al.’s [28] method, with clearer outlines in their reconstructions, obtains the best results on the SSIM metrics. As our approach emphasizes reconstruction matching the semantic content of the perceptual image, it may lag behind approaches that emphasize pixel similarity in terms of low-level metrics (Shen et al. [26], Beliy et al. [27], Gaziv et al. [28], and Ren et al. [29]). However, DBDM excels in achieving the best performance on all high-level metrics. It is noteworthy that our method outperforms other semantic-focused approaches (Ozcelik et al. [31], Mozafari et al. [13], and Liu et al. [20]) in both low-level and high-level metrics. In particular, compared to the method that also uses a diffusion model for visual reconstruction (Liu et al. [20]), DBDM improves the low-level assessment metrics PixCorr and SSIM by 32% and 5.6%, respectively, and reduces the Inception distance, CLIP distance, and SwAV distance by 32.7%, 25.2%, and 23.1%, respectively. This superiority is further underscored by quantitative comparisons, highlighting the efficacy of our proposed method.

### 3.4. Ablation Studies

In this section, we conduct ablation experiments to investigate the individual contributions of different components in the proposed model. The quantitative comparison results are presented in Table 2.

**Figure 5:**
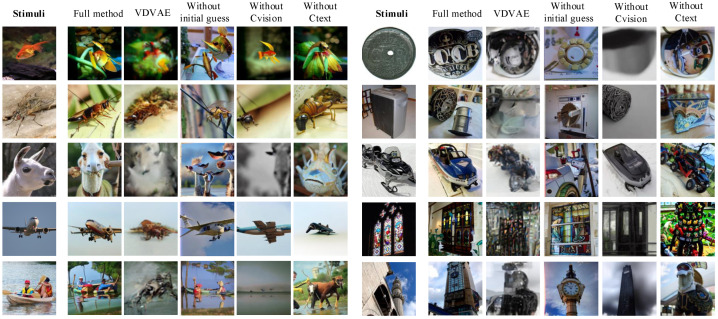
Examples of reconstructed images from ablation studies, all from subject 3.

Several interesting findings emerge from the experimental results. When the initial guess image is not utilized, the reconstruction performance of the model is inferior in terms of low-level metrics (PixCorr and SSIM declined by 41.9% and 18.1%, respectively) but relatively strong in high-level metrics. This suggests that the introduction of an initial guess image in DBDM facilitates the preservation of underlying details from the original image, thereby enhancing reconstruction performance. However, even in the absence of an initial guess, our model can accurately capture the semantic content of the stimulus images. For the example of the airplane in Figure 5, DBDM still accurately reconstructed the image containing the airplane object when the initial guess image was missing. Moreover, when there is no semantic guidance (without CLIP text), the high-level metrics of the reconstructed images experience a significant decline; Inception distance, CLIP distance and SwAV distance increased by 0.229, 0.171 and 0.168, respectively. This underscores the importance of semantic information in optimizing the quality of reconstructions. Similarly, when visual guidance is omitted, the high-level metrics of the reconstructions also decrease, since CLIP visual features inherently encompass the semantic content of the original images. Surprisingly, the model without CLIP visual achieves the best performance in low-level metrics. This discrepancy may be attributed to limitations in the diffusion process, resulting in the retention of most of the details from the initial guesses in the reconstructed images (Figure 5). Overall, the most effective performance is achieved when employing the full model.

For visual comparison, we present our qualitative results in Figure 5. The reconstructed images using VDVAE retain the layout of the original images but appear blurry and challenging to identify. The reconstructed images without CLIP text struggle to capture the semantic information of the objects in the stimulus images. Additionally, we observed that the partially reconstructed images without CLIP visual resemble the initial guesses, and some even exhibit blurriness. This can be seen, for example, in the horse in the third row of Figure 5.

### 3.5. Effectiveness of BrainMlp for Neural Decoding

In this section, we examine the efficacy of using BrainMlp as a neural decoder for decoding feature vectors from fMRI patterns. Specifically, we employ the traditional ridge regression model to replace BrainMlp, wherein VDVAE-regression, CLIP text regression, and CLIP vision regression refer to the use of ridge regression to predict the corresponding $z_{test}$, ${\hat{c}}_{text}$, and ${\hat{c}}_{vision}$ from the test fMRI data, respectively. The results in Table 3 demonstrate that BrainMlp outperforms the simple ridge regression model in learning the mapping from the fMRI data to the image feature space owing to the complexity of the brain’s visual encoding mechanism. For instance, the initial reconstructions obtained using BrainMlp to predict VDVAE encoder hierarchical features exhibit superior low-level metrics compared to the simple regression model (PixCorr: 0.254 vs. 0.213; SSIM: 0.447 vs. 0.434). Moreover, when employing the regression model instead of BrainMlp to predict ${\hat{c}}_{text}$ and ${\hat{c}}_{vision}$ from the fMRI patterns, both high-level metrics of the reconstructed images experience a significant decline. Among them, the CLIP text regression model demonstrates a more pronounced performance drop, with Inception distance, CLIP distance, and SwAV distance increasing by 23.3%, 40.9%, and 20.9%, respectively, emphasizing the substantial influence of accurate semantic decoding on the reconstruction quality of DBDM. This further substantiates the effectiveness of employing BrainMlp as a neural decoder, which greatly impacts the performance of our model.

## 4. Discussion

In this study, we introduced the Dual-guided Brain Diffusion Model (DBDM) to address the challenge of reconstructing visual stimuli from fMRI signals. By dividing the visual reconstruction process into three stages, we aimed to gradually recover the visual information perceived by the brain. In the first stage, we employed VDVAE to generate coarse reconstructions of the visual stimuli. Subsequently, in the second stage, we utilized BLIP to obtain text annotations for each image, which were used to extract semantic features in the next stage. Finally, in the third stage, we used the predicted CLIP vision and CLIP text features by BrainMlp as conditional bootstraps to guide the diffusion model in generating the final reconstructions.

We performed qualitative (Figure 3) and quantitative analyses (Table 1) of the reconstruction results. Figure 3 illustrates that the reconstructed images produced by our model may not be identical to the original images, but they successfully retain the primary semantic content and preserve most pixel-level details of the original images. This aligns with the emphasis of our method on recovering the semantic content of the perceptual images. Compared to previous methods that emphasize pixel similarity, DBDM-reconstructed images are more natural, while for methods that focus on semantic restoration, DBDM reconstructions have more consistent semantics with the original images (Figure 4). In the quantitative comparisons, although our approach may not perform as strongly as methods that focus solely on pixel space similarity in low-level metrics, our reconstructions consistently outperform them in high-level metrics and surpass other semantic-focused methods. In particular, DBDM reduced the Inception distance, CLIP distance, and SwAV distance by 32.7%, 25.2%, and 23.1%, respectively, compared to the SOTA reconstruction approach (Liu et al. [20]). The reason why our method outperforms previous models is mainly attributed to the following facts: (1) we employed the Versatile Diffuser (VD) with its powerful generative capabilities as a generator; (2) we used VDVAE to reconstruct the initial guess image to capture the underlying details of the original image; (3) we constructed semantic annotations for the images using BLIP and double-conditioned the inverse diffusion process using the CLIP vision and CLIP text features; and (4) we used the construction of neural decoders with residual connections to learn the mapping of fMRI data to visual and semantic features.

Notably, our reconstructed images have higher resolution compared to previous low-quality reconstructions owing to the powerful generative capability of the latent diffusion model. This observation inspires us to explore more powerful deep generative models that can potentially lead to even higher quality reconstructions. We are optimistic that, with the continued advancement of generative models, visual reconstruction techniques will improve significantly and achieve remarkable levels of accuracy and fidelity. Regarding fMRI decoding, our experiments reveal that using a deep neural network with residual connections, such as BrainMlp, outperforms traditional ridge regression models. BrainMlp is able to learn the complex mapping between fMRI signals and deep neural network features more effectively without encountering the issue of overfitting. This highlights the potential of utilizing advanced neural decoding techniques to enhance the performance of fMRI-based reconstructions.

However, there are still areas that require further refinement in our model. For instance, the current BrainMlp model may not accurately predict the expected feature vectors from fMRI data, resulting in imperfect replications of the original stimulus images. As seen in Figure 3, the model may successfully reconstruct an airplane, but the image may not precisely match what the subject actually saw. Such discrepancies could be attributed to inherent variations in the way subjects perceive and process stimuli. To improve the accuracy of fMRI decoding, acquiring more paired samples and higher signal-to-noise ratio fMRI data becomes imperative. Unfortunately, fMRI data acquisition is time-consuming and costly, making it challenging to obtain sufficient samples for comprehensive training. Additionally, training different decoders for each subject, as done in our study due to the varying dimensions of fMRI data, can lead to redundancy and inefficiency. As such, developing a neural decoder that can generalize across different subjects would be a valuable avenue of research.

Furthermore, with the advancement of neural decoding techniques, ethical considerations must be addressed. The application of image reconstruction methods has the potential to raise privacy concerns, as it could be used to invade the privacy of individuals or create misleading content. As this technology progresses, responsible and transparent practices should be adhered to in order to ensure the ethical use of such tools.

## 5. Conclusions

In this paper, our proposed Dual-guided Brain Diffusion Model (DBDM) presents a promising solution for reconstructing visual stimuli from fMRI data. By dividing the reconstruction process into three phases and leveraging image generation models, DBDM effectively captures semantic content and preserves pixel-level details. Our experimental results demonstrate superior performance in high-level metrics compared to pixel similarity-focused methods and other semantic-focused approaches. Despite its strengths, improvements are needed in fMRI decoding accuracy, and ethical considerations must be taken into account for responsible use. With continued research, DBDM holds potential for advancing neuroscience and related fields by enhancing our understanding of the human brain’s visual perception.

## Figures and Tables

**Table 1:** Quantitative evaluation of the reconstructed image quality, which is calculated using the images in Figure 4. For low-level evaluation metrics (PixCorr and SSIM), the higher their values, the better. For high-level semantic metrics (Inception, CLIP, and SwAV distances), the lower their values, the better. The best results are shown in bold.

Methods	Quantitative Measures				
	Low-Level↑		High-Level↓		
	PixCorr	SSIM	Inception Distance	CLIP Distance	SwAV Distance
Beliy et al., 2019 [27]	0.351	0.575	0.896	0.415	0.690
Gaziv et al., 2022 [28]	0.459	**0.607**	0.871	0.389	0.592
Ozcelik et al., 2022 [31]	0.223	0.453	0.846	0.340	0.510
Mozafari et al., 2020 [13]	0.103	0.431	0.932	0.346	0.577
Ren et al., 2021 [29]	**0.657**	0.605	0.838	0.393	0.617
Shen et al., 2019 [26]	0.339	0.539	0.933	0.379	0.581
Liu et al., 2023 [20]	0.175	0.448	0.908	0.301	0.527
Ours	0.231	0.473	**0.611**	**0.225**	**0.405**

**Table 2:** DBDM ablation studies. All reported results were calculated on the 50 reconstructed images of Subject 3, and the best results are bolded.

Model	Quantitative Measures				
	Low-Level↑		High-Level↓		
	PixCorr	SSIM	Inception Distance	CLIP Distance	SwAV Distance
without initial guess	0.136	0.316	0.789	0.337	0.522
without CLIP-text	0.222	0.376	0.977	0.476	0.636
without CLIP-vision	**0.248**	**0.446**	0.827	0.345	0.542
full method	0.234	0.386	**0.748**	**0.305**	**0.468**

**Table 3:** Quantitative comparisons between BrainMlp and ridge regression in terms of reconstructed image quality. All reported results were calculated on 50 reconstructed images using the test data from subject 3. The best results are shown in bold.

Model	Quantitative Measures				
	Low-Level↑		High-Level↓		
	PixCorr	SSIM	Inception Distance	CLIP Distance	SwAV Distance
CLIP text regression	0.204	0.348	0.922	0.430	0.566
CLIP vision regression	0.222	0.357	0.880	0.398	0.536
VDVAE regression	0.213	0.434	0.962	0.433	0.644
VDVAE–BrainMlp	**0.254**	**0.447**	0.960	0.435	0.631
full method	0.234	0.386	**0.748**	**0.305**	**0.468**

## Data Availability

The fMRI data used in this research are publicly available at [11].
